# Supplementary material for: A Critical View of the Application of the APEX Software (Aqueous Photochemistry of Environmentally-Occurring Xenobiotics) to Predict Photoreaction Kinetics in Surface Freshwaters
Source: Molecules. 2019 Dec 18;25(1):9. doi: 10.3390/molecules25010009 (PMC7017383; doi:10.3390/molecules25010009)
Supplement: Supplementary file 1 [file molecules-25-00009-s001.zip › 3.2.4_gcc-4.4.0.zip!/3.2.4_gcc-4.4.0/3.2.4_gcc-4.4.0/doc/octave/html/interpreter/Calling-Octave-Functions-from-Oct_002dFiles.html]

Calling Octave Functions from Oct-Files - Untitled


Next: Calling External Code from Oct-Files,
Previous: Accessing Global Variables in Oct-Files,
Up: Oct-Files


---

#### A.1.8 Calling Octave Functions from Oct-Files

There is often a need to be able to call another octave function from
within an oct-file, and there are many examples of such within octave
itself. For example the `quad` function is an oct-file that
calculates the definite integral by quadrature over a user supplied
function.

There are also many ways in which a function might be passed. It might
be passed as one of

1. Function Handle- Anonymous Function Handle- Inline Function- String

The example below demonstrates an example that accepts all four means of
passing a function to an oct-file.

```
```
     #include <octave/oct.h>
     #include <octave/parse.h>
     
     DEFUN_DLD (funcdemo, args, nargout, "Function Demo")
     {
       int nargin = args.length();
       octave_value_list retval;
     
       if (nargin < 2)
         print_usage ();
       else
         {
           octave_value_list newargs;
           for (octave_idx_type i = nargin - 1; i > 0; i--)
             newargs (i - 1) = args(i);
           if (args(0).is_function_handle ()
               || args(0).is_inline_function ())
             {
               octave_function *fcn = args(0).function_value ();
               if (! error_state)
                 retval = feval (fcn, newargs, nargout);
             }
           else if (args(0).is_string ())
             {
               std::string fcn = args (0).string_value ();
               if (! error_state)
                 retval = feval (fcn, newargs, nargout);
             }
           else
             error ("funcdemo: expected string,",
     	       " inline or function handle");
         }
       return retval;
     }
```
```

The first argument to this demonstration is the user supplied function
and the following arguments are all passed to the user function.

```
     funcdemo (@sin,1)
     => 0.84147
     funcdemo (@(x) sin(x), 1)
     => 0.84147
     funcdemo (inline ("sin(x)"), 1)
     => 0.84147
     funcdemo ("sin",1)
     => 0.84147
     funcdemo (@atan2, 1, 1)
     => 0.78540
```

When the user function is passed as a string, the treatment of the
function is different. In some cases it is necessary to always have the
user supplied function as an `octave_function` object. In that
case the string argument can be used to create a temporary function like

```
     std::octave fcn_name = unique_symbol_name ("__fcn__");
     std::string fname = "function y = ";
     fname.append (fcn_name);
     fname.append ("(x) y = ");
     fcn = extract_function (args(0), "funcdemo", fcn_name,
                             fname, "; endfunction");
     ...
     if (fcn_name.length ())
       clear_function (fcn_name);
```

There are two important things to know in this case. The number of input
arguments to the user function is fixed, and in the above is a single
argument, and secondly to avoid leaving the temporary function in the
Octave symbol table it should be cleared after use.
